# Comparison of Subtypes of *Listeria monocytogenes* Isolates from Naturally Contaminated Watershed Samples with and without a Selective Secondary Enrichment

**DOI:** 10.1371/journal.pone.0092467

**Published:** 2014-03-20

**Authors:** Lisa Gorski, Samarpita Walker, Anita S. Liang, Kimberly M. Nguyen, Jessica Govoni, Diana Carychao, Michael B. Cooley, Robert E. Mandrell

**Affiliations:** Produce Safety and Microbiology Research Unit, United States Department of Agriculture, Agricultural Research Service, Albany, California, United States of America; St. Petersburg Pasteur Institute, Russian Federation

## Abstract

Two enrichment methods for *Listeria monocytogenes* using Immuno Magnetic Separation (IMS) were tested to determine if they selected the same subtypes of isolates. Both methods used a non-selective primary enrichment and one included subculture in Fraser Broth, while the other involved direct plating of IMS beads. Sixty-two naturally contaminated watershed samples from the Central California Coast were used as a source of *L. monocytogenes*, and subtype diversity was measured by serotype and Multiple Number Variable Tandem Repeat Analysis (MLVA). Three different serotypes were detected from both methods with serotype 4b strains making up 87% of the isolates, serotype 1/2a making up 8%, and serotype 1/2b making up 5%. The data suggest that serotype 1/2a strains were more likely to be isolated from the Fraser Broth culture method. Sixty-two different MLVA types were detected and the more common MLVA types were detected by both culture methods. Forty-three MLVA types were detected only from one culture method or the other, while 19 types were detected from both culture methods. The most common MLVA type-12 was detected in 33 of the 62 water samples, and represented 31% of the isolates from both culture methods. This limited study provides evidence that using both enrichment culture methods allowed for detection of a greater diversity of isolates among the samples than the use of one method alone, and that a wide diversity of *L. monocytogenes* strains exist in this watershed.

## Introduction

*Listeria monocytogenes* is a gram-positive facultatively intracellular, foodborne pathogen that survives in nature as a saprophyte. Between 2009–2011 there were 1651 cases of listeriosis in the United States with a fatality rate of 21%, with the majority of cases in elderly and immunocompromised individuals, and 14% of the cases pregnancy-related. In the environment *L. monocytogenes* survives in agricultural niches, wildlife, soil, and water [1]–[5]. Soft cheeses and ready-to-eat meats are commonly associated with outbreaks, but in recent years two high profile outbreaks have been linked to raw produce [6], [7].

Several methods are used for the enrichment and isolation of *Listeria monocytogenes*. Many of these methods utilize selective compounds such as dyes and antibiotics to favor *L. monocytogenes* over competing microbiota. Studies indicate that these enrichment methods show bias in the types of *Listeria* spp. and/or *L. monocytogenes* strains that are isolated as a result of different strains outcompeting others in selective enrichment cultures [8]–[11]. For this reason non-selective media are sometimes used for part or all of the enrichment process. Immunomagnetic separation (IMS) using magnetic beads containing antibodies specific for the target organism is a convenient tool for non-selective enrichments [12], [13]. Nevertheless, there is always a question with enrichment culture whether the isolates represent an accurate reflection of what is present in an environment. This can be a problem particularly in traceback investigations. Our goal to detect strains of *L. monocytogenes* present in a produce production environment watershed stimulated comparing methods of enrichment and the characteristics of *L. monocytogenes* strains isolated by different media. A primary objective was to determine if the enrichment media affected the types of *L. monocytogenes* that were isolated.

We used a non-selective enrichment broth followed by IMS for the isolation of *L. monocytogenes* from Moore swab samples taken from a naturally contaminated watershed on the Central California coast, and compared a secondary selective enrichment with direct plating onto selective and indicator medium. We wanted to determine if the isolates obtained from the same samples via selective and non-selective media were the same subtypes. Serotype analysis is one of the most important subtyping methods used for *L. monocytogenes*, but it provides limited discrimination. Pulsed field gel electrophoresis is widely used for subtyping, but it is time consuming [14]. Multilocus Tandem Variable Repeat Analysis (MLVA) is a faster throughput method that measures the number of tandem repeats present in a number of different loci on the chromosome, and several MLVA methods for *L. monocytogenes* have been developed in the last few years [15]–[17]. The isolates in the present study were subtyped by serotype analysis and MLVA.

## Materials and Methods

### Sampling and Enrichment

Moore swabs (cut cotton gauze tied to a string) were deployed in lakes, rivers, streams, and ponds in Monterey County for up to 24 hours as described previously [18]. Since all of the sampling sites were on public lands there were no specific permissions required for sampling. None of the studies involved endangered or protected species, and all sampling occurred in an approximate 20 mile radius of Salinas, CA. Swabs were transported to the lab on the day of collection, and were rinsed with 0.5 L of sterile water. One hundred ml of rinsate was measured into a 500-ml Whirl-pak bag to be used for *L. monocytogenes* enrichment. Twenty-five ml of Buffered *Listeria* Enrichment Broth Base (BLEB, Difco, Becton-Dickinson-BBL, Franklin Lakes, NJ; 5X manufacturer’s instructions) made at a 5X concentration were added to the 100 ml of rinsate, and the bags were incubated on a rotating shaker at 150 rpm at 30°C for 18 hours, and then held at 4°C until the following morning (usually 1–4 hours). Anti-*Listeria* immunomagnetic beads (Dynal, Life Technologies, Grand Island, NY) were added to the enrichment cultures (20 μl of beads into 1 ml of culture) and the cell-bead suspensions were incubated, rinsed, and collected in a bead retriever using manufacturer’s instructions (Dynal). Beads were collected into 130 μl of Phosphate Buffered Saline with Tween (PBS-Tween: 10 mM sodium phosphate, pH 7.2, 150 mM NaCl, 0.05% Tween-20).

After bead collection 30 μl were deposited onto a Brilliance *Listeria* Agar plate (Oxoid, Remel, Lenexa, KS) and streaked to obtain isolated colonies, and the plates incubated at 37°C for 2 days. The remaining 100 μl of bead suspension were inoculated into 5 ml of Fraser broth (Difco) supplemented with Ferric Ammonium Citrate (0.5 g/L), and incubated at 37°C for two days. Thirty microliters of black Fraser broth cultures were inoculated onto Brilliance *Listeria* agar plates, and streaked for isolated colonies. Plates were incubated for 2 days at 37°C. Fraser broth cultures that failed to turn black were discarded. Up to three colonies that were blue with a zone of clearing on Brilliance plates were picked, deposited onto Trypticase Soy Agar plates (TSA, Difco), and grown overnight at 37°C. Isolates were further cleaned by culturing on Modified Oxford Agar (MOX, Difco), which was incubated at 37°C. Isolated bluish-white colonies with a zone of black precipitate on MOX were streaked onto TSA for further analysis. Hemolysis was tested by depositing isolates onto TSA containing 5% sheeps blood (Remel), and incubating up to 5 days at 37°C. Clearing underneath colonies could be discerned by holding the plates up to a light to determine transparency.

### Confirmation of *L. monocytogenes* and Serotyping

Isolates were screened by PCR for the *hlyA* gene using the primers and protocol of Norton *et al.* [19] using One*Taq* Hot Start (New England Biolabs, Ipswich, MA), and the PCR products were electrophoresed on a 1% agarose gel stained with GelRed (Phenix Research Products, Candler, NC). PCR template was made by picking a piece of colony with a sterile, disposable needle and depositing it into a PCR tube that contained 50 μl of 1X PCR buffer and 50 μl of 1% Triton X-100. These tubes were then incubated in a thermocycler for 15 min at 100°C, and after they had cooled, 5 μl of the cell suspension was used as PCR template. Isolates that had a 858 bp amplicon were considered tentatively positive and were serotyped for O-antigen by an ELISA serotyping method [20]. Any isolate that was not clearly designated as serotype 1/2, 3, or 4 by O-typing sera were discarded from further analysis, as this encompasses 12 of the 13 known *L. monocytogenes* serotypes except for the extremely rare serotype 7. ELISA serotyping using O-antigen antisera is sufficient to determine any serotype 4 isolate. Isolates that were O-serotype 1/2 or 3 were further screened for H-antigen using the multiplex PCR serotyping method of Doumith *et al.* [21].

### Determination of MLVA Profiles

Genomic DNA was extracted using the Wizard Genome Purification Kit (Promega, Madison, WI). Cultures were grown in TSB overnight at 37°C, and 1.3 ml was centrifuged for 5 minutes and the pellets resuspended in TE (Tris-HCl, pH 8.0, 1 mM EDTA) containing 10 mg/ml lysozyme, and the suspensions incubated at 37°C for 30–45 minutes. The suspension was pelleted by centrifuging at 13,000 rpm for 5 min and the pellet then lysed and treated following manufacturer’s instructions for genomic DNA purification.

MLVA analysis was conducted by the method of Sperry *et al*. [15], [22] with some modifications. The primers sequences (Table 1) were the same as used in the original Sperry *et al.* and the addendum to that paper that corrected the sequence of Lm-23 reverse primer [15], [22]; however, different dyes were incorporated into the primers because different equipment (detailed below) was used in the current study. Primers and dyes were purchased from Applied Biosystems (Life Technologies, Grand Island, NY). Multiplex PCR was done as described [15] with separate R1 and R2 reactions using the Type-it Microsatellite PCR Kit (Qiagen, Valencia, CA). The resulting PCR products were diluted 1∶60 in sterile water, and fragments were sized by mixing 1 μl of diluted PCR product, 18.5 μl of deionized formamide, and 0.5 μl of GeneScan 600 LIZ Size Standard (Applied Biosystems). Fragments were separated using an Applied Biosystems 3130*xl* Genetic Analyzer that was calibrated with Matrix Standard Kit Dye Set D (Applied Biosystems); and the fragments sizes were determined with GeneMarker software v. 2.4 (Soft Genetics, State College, PA). Fragment sizes, peak heights, and dye color were imported into BioNumerics v. 6.6 (Applied Maths, Austin, TX). Variable Number Tandem Repeat (VNTR) parameters were inputted into BioNumerics as reported [15], but offsets were adjusted because of equipment and reagent differences from those previously reported (K. Sperry, personal communication). Offsets were calculated by determining the MLVA profiles of *L. monocytogenes* strains F6854, F6900, J0161, G3984, F2365, L4738, and EGDe, and adjusting the offsets until all of the VNTR loci agreed with previously published values [15]. BioNumerics parameters used to assign the copy number of VNTR loci are shown in Table 2. The minimum value for the Lm-32 locus was adjusted to 10 from 13. Null alleles were coded as negative. A categorical similarity matrix was generated in BioNumerics using the unweighted pair group method with arithmetic averages (UPGMA). BioNumerics assigned a value of −2 to loci for which there was no peak. A categorical tree was constructed and MLVA types were assigned arbitrarily based on the similarity matrices. Each of the isolates of an MLVA type has 100% similarity to each other in the number of VNTR copies. A minimal spanning tree for categorical data to compare Fraser Broth and non-Fraser broth isolates was constructed using UPGMA and the default categorical coefficient to calculate the distance matrix. Simpson’s index of diversity was calculated as described [23].

**Table 1:** Primers and Dyes used for MLVA analysis.

PCR	VNTR locus	Forward primer (5′ → 3′)^a^	Reverse primer (5′ → 3′)
R1	Lm-2	(6-FAM)-CGTATTGTGCGCCAGAAGTA	CAGCAACGCAACAACAAACAG
	Lm-8	(NED)-ACGCGCAATACTATAAAGGGTGTC	AGAAAAAGCGGAAGCAGATAAGAA
	Lm-10	(VIC)-CAGATATCGATACGATTGAC	CAGTTAGTATTTCCAACGTC
	Lm-11	(NED)-GAATAAAATGCTAGATGTGG	CCGATTCAAAAATAGTAAAC
R2	Lm-3	(6-FAM)-CAAACCGAGATGGTGTAGCA	TGGTTTTGATGGATCAACTGG
	Lm-15	(NED)-GGACTTAACGAATACAAAAG	GCTGTTACAAGTAAAACTGG
	Lm-23	(VIC)-TACGCCAGTTCCTCCGTTAG	TTGAAAGCTGGAGATGTTA
	Lm-32	(VIC)-AAAGCTTTGCCAGTGCAAGT	TTGTGACTTGGCACTTCTGG

a Dye is shown in parentheses before primer sequence.

**Table 2:** Parameters for *L. monocytogenes* VNTR Loci.

Name	Reaction	Dye Type^a^	Offset	Length	Min value	Max value	Tolerance
Lm-2	R1	6-FAM	287	6	11	20	3
Lm-8	R1	NED	185	15	3	4	7
Lm-10	R1	VIC	283	12	3	9	6
Lm-11	R1	NED	102	12	1	6	6
Lm-3	R2	6-FAM	197	9	1	9	4
Lm-15	R2	NED	316	12	1	7	6
Lm-23	R2	VIC	72	6	15	42	3
Lm-32	R2	VIC	80	6	10	21	3

a 6-FAM: blue, VIC: green, NED: yellow.

## Results

### Isolation and Serotypes of *L. monocytogenes*

There were 206 Moore swab samples collected from 30 different locations among rivers, lakes, and streams at 8 different sampling dates between January and March 2012. Sixty-two of those samples from 24 different locations yielded *L. monocytogenes* isolates from both direct plating of IMS beads (D-isolates) and Fraser Broth-enriched IMS beads (F-isolates). The 304 individual isolates resulting from those 62 Moore swab samples were compared to determine if the 2 culturing methods were enriching the same molecular subtypes. Characteristics of each individual isolate are given in Supporting Table S1. A summary of the samples and the characteristics of the isolates obtained from them are shown in Table 3. Three isolates from each Brilliance plate were selected, but not every isolate was confirmed as *L. monocytogenes*. The 62 Moore swab samples gave a total of 304 individual isolates with 157 D isolates, and 147 F isolates. All but 14 isolates showed lytic activity on blood agar plates. Table 4 summarizes the serotypes recovered from the 304 individual isolates. Only 3 serotypes were detected among the isolates with 87% belonging to serotype 4b, and 8% and 5% belonging to serotypes 1/2a and 1/2b, respectively (Table 4 and Supporting Table S1). The discriminatory index based on serotyping was higher from Fraser broth than from Direct plating, indicating that a greater diversity of serotypes were found from the Fraser Broth culturing, but in all cases with serotyping this index was less than 0.3, which was not very discriminatory (Table 4). However, 11.6% of the isolates obtained through the Fraser broth culturing method were of serotype 1/2a, as compared to 5.1% from the Direct plating method. In contrast 90% of the isolates from the Direct plating method were of serotype 4b, as compared to 83% from Fraser broth. These data suggest that while serotyping is not very discriminatory in measuring the diversity in an environment in general, multiple culturing methods allow for a greater assortment of serotypes to be isolated. Multiple serotypes were detected from 15 Moore swab samples; however, for 14 of those samples the additional serotypes were detected only from one of the culturing methods. These multiple serotypes would not have been identified if only one culturing method were used.

**Table 3:** Characterization of isolates from samples.

Sample ID	No. of D isolates	D serotypes (MLVA types)^a^	No. of F isolates	F serotypes (MLVA types)^a^
131	1	4b (9)	3	1/2a (49)
132	3	4b (56), 1/2a (57)	1	1/2a (56)
136	3	4b (12)	3	4b (12)
137	2	4b (6)	3	4b (5,6)
138	2	4b (9, 12)	3	4b (9,12)
139	3	4b (12)	3	1/2a (54)
143	3	4b (6)	3	4b (6)
157	1	4b (55)	3	1/2a (60), 4b (55)
158	3	4b (27, 59)	2	1/2a (52, 59)
162	2	1/2b (1)	1	1/2b (1)
164	3	4b (12, 36)	3	1/2a (54), 1/2b (12), 4b (12)
177	3	4b (17)	2	4b (3, 17)
191	3	4b (4, 19, 43)	3	4b (19)
192	3	4b (12, 13, 17)	3	4b (12, 27)
195	3	4b (22, 23)	1	4b (17)
200	2	4b (17)	2	4b (17, 30)
202	3	4b (2, 19)	3	4b (12, 15, 26)
208	2	4b (18)	3	4b (3,12)
213	3	1/2a (42)	3	1/2a (42, 45)
218	2	4b (12, 14)	3	4b (11,12)
219	3	1/2b (48), 4b (9)	3	4b (9)
220	3	1/2a (50), 4b (50)	3	4b (12, 54)
224	2	4b (17, 36)	1	4b (17)
226	1	4b (6)	2	4b (5, 6)
229	3	4b (12, 36)	3	4b (9,12 37)
240	2	4b (9, 12)	3	4b (27)
242	3	4b (12, 19, 26)	2	4b (12)
243	3	4b (17)	3	4b (12)
245	3	4b (17)	3	4b (11, 12)
246	3	4b (12, 35)	2	4b (12)
250	3	4b (12, 50)	3	4b (14, 16, 17)
251	3	4b (12)	3	4b (16, 17)
259	3	4b (10, 12)	3	4b (17, 19)
262	3	4b (19)	3	4b (19)
266	3	4b (12)	3	4b (12)
267	3	4b (14)	4	4b (14)
269	2	4b (12)	3	4b (12)
270	3	4b (12)	3	4b (12, 34)
278	3	4b (36, 40)	3	4b (36, 38)
288	1	4b (6)	2	4b (6, 62)
290	3	4b (19)	1	4b (6)
291	3	4b (12)	1	1/2b (41)
292	3	4b (12, 28, 53)	3	1/2b (12, 33)
293	3	4b (9)	2	4b (53)
295	3	4b (12, 29)	3	4b (31, 32, 39)
297	3	4b (35, 36)	1	4b (12)
298	3	1/2a (46), 1/2b (3), 4b (47)	1	4b (36)
300	2	4b (9, 21)	1	4b (61)
304	3	1/2b (8), 4b (3, 34)	3	4b (12, 61)
305	1	1/2b (17)	2	4b (6,12)
309	2	4b (20, 24)	2	4b (25)
313	2	4b (17)	2	4b (17)
314	2	1/2a (44), 4b (58)	3	1/2a (58), 4b (58)
315	1	4b (19)	2	1/2a (51), 4b (19)
316	2	4b (6)	2	4b (6, 19)
319	3	4b (12)	2	4b (12)
321	1	4b (9)	1	4b (9)
323	2	4b (12)	2	4b (12)
324	3	4b (12, 36)	1	4b (12)
326	3	4b (6, 12, 36)	3	4b (7,12)
327	3	4b (7, 12, 36)	1	4b (54)
331	3	4b (12, 27)	2	4b (12)

a Number in parentheses refers to the assigned MLVA type number.

**Table 4:** Numbers of isolates of each serotype and the Diversity index for serotyping and for MLVA types in the samples based on the enrichment methods.

Method	Serotype1/2a	Serotype1/2b	Serotype4b	Simpson’s Index ofDiversity (Serotypes)	Number ofMLVA Types	Simpson’s Index ofDiversity (MLVA Types)
Directplating	8	7	142	0.179	41	0.875
Fraserbroth	17	7	123	0.286	40	0.887
Total	25	14	265	0.232	62	0.881

### MLVA Typing

**Figure 1:**
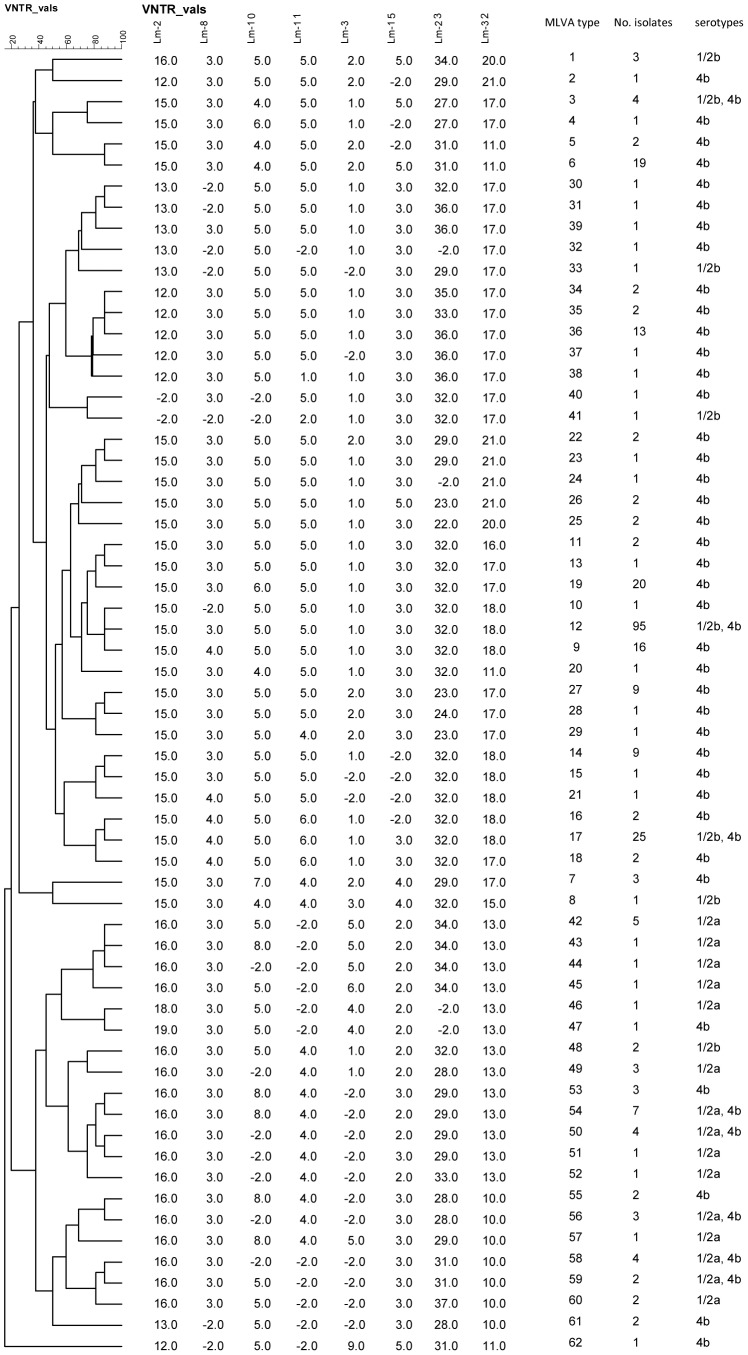
Categorical tree of MLVA types based on differences in VNTR copy number, and the numbers of isolates of each type and the serotypes represented in them. BioNumerics assigns a value of −2 to loci yielding no product.

Sixty-two different MLVA types were detected among the 304 isolates. The evolutionary relationships between the MLVA types and the numbers of isolates in each type are shown in the categorical comparison tree in Figure 1. MLVA type-12 was detected most frequently with 95 isolates from 33 Moore swab samples at 16 different locations and 8 different sampling dates. MLVA type-17 was the second most common with 25 isolates from 13 Moore swab samples at 9 different locations and 6 different sampling dates. Four of the 14 non-hemolytic isolates were located in the clade in Figure 1 encompassing MLVA subtypes 30, 31, 32, 33, 39; three non-hemolytic isolates were of MLVA type-12; and the remaining were scattered (1 each of types 3, 20, 21, 36, 40, 61, and 62). Some of the MLVA types contained more than one serotype. MLVA types 3, 12, and 17 contained isolates of 1/2b and 4b; while MLVA types 50, 54, 56, 58, and 59 contained isolates of 1/2a and 4b. The MLVA type of each isolate is shown in Table 3 in parentheses after the serotype.

**Figure 2:**
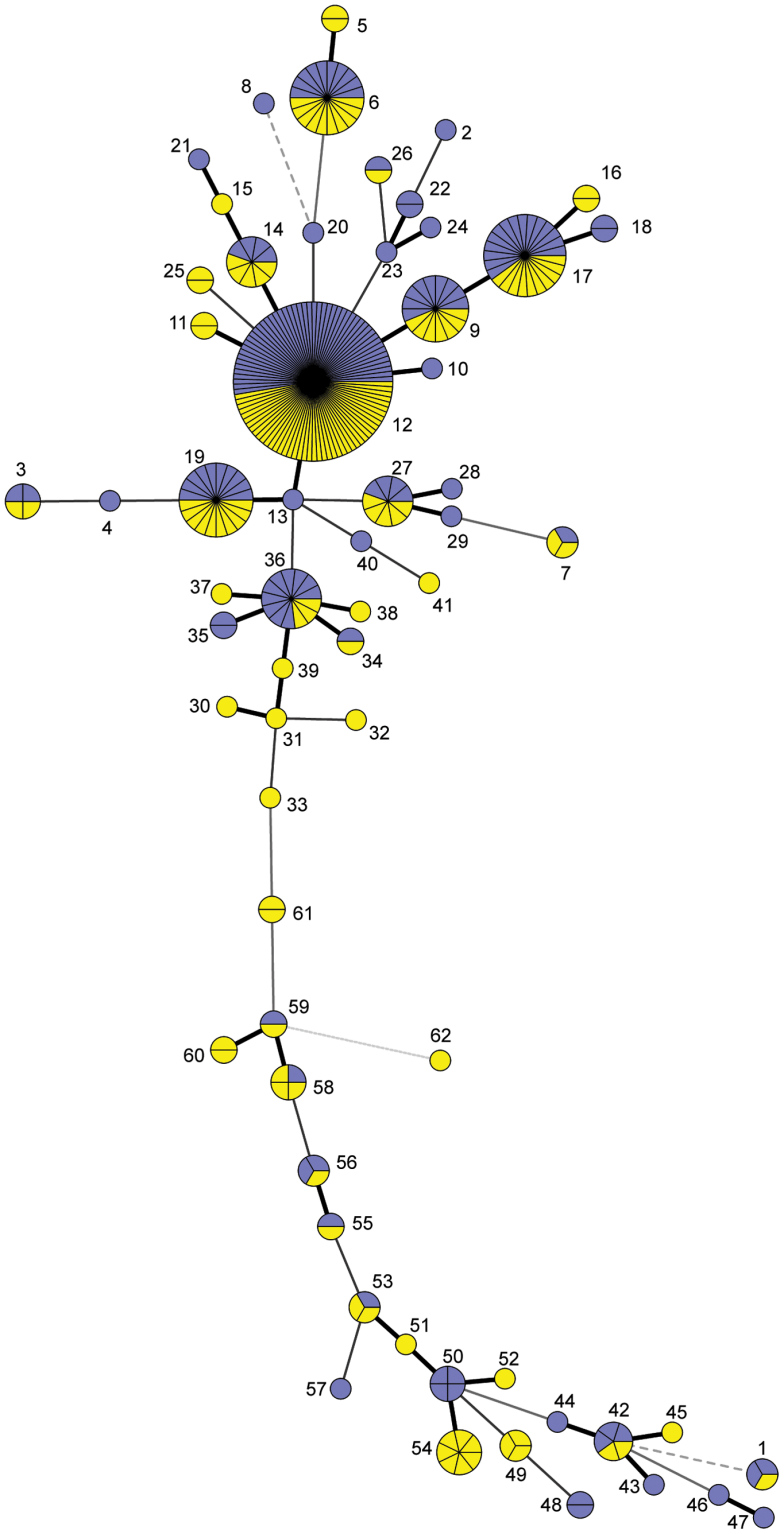
Minimum spanning tree with categorical data of all 62 MLVA types containing all 304 isolates. Each circle is labeled with the MLVA type it represents. The size of the circle and number of divisions represent the number of isolates of that type. The blue and yellow segments represent isolates obtained by Direct plating or with Fraser broth, respectively. Heavy short connecting lines indicate a difference in a single locus; thinner, longer lines connect nodes with 2 and 3 locus differences; dotted lines connect nodes with 4 locus differences; a gray line connects nodes with 5 locus differences.

A minimum spanning tree of all of the isolates and their MLVA types was constructed and differentiated by D and F isolates (Figure 2). The tree shows that 19 MLVA types, including the largest groupings, were detected from both types of enrichments. The diversity indices for the methods based on MLVA are shown in Table 4. The index for the individual methods and both together were similar and ranged from 0.875–0.887, indicating that both methods reflected a wide diversity of MLVA types in the region. However, using both culturing methods allowed more MLVA types to be detected. Fifty-one Moore swab samples contained more than one MLVA type. Figure 2 illustrates that 43 MLVA types were found only among either D isolates (22 types) or F isolates (21 types), and 30 MLVA types were detected only once (17 among D isolates, 13 among F isolates).

We were interested in the ability of the different culture methods to isolate the same MLVA types from different Moore swab samples, which would be important in assessing ecology in a region. Of the 304 isolates 236 belonged to MLVA types that were detected from more than one Moore swab sample (Table 5). Most of these MLVA types were isolated from both D and F methods; however, types 5, 11, 16, 54, and 61 were isolated only with Fraser broth, and type-50 isolates were recovered only through direct plating.

**Table 5:** MLVA types detected from more than one water sample and enrichment type from which they were isolated.

MLVA type	Number of isolates (n = 304)	Number of samples (n = 62)	Plating type
12	95	33	Both
17	25	13	Both
6	20	8	Both
19	20	8	Both
9	16	8	Both
36	13	9	Both
14	9	4	Both
27	9	4	Both
54	7	4	F only
3	4	4	Both
50	4	2	D only
7	3	2	Both
53	3	2	Both
5	2	2	F only
11	2	2	F only
16	2	2	F only
61	2	2	F only

## Discussion

Bacteria in nature and in foods often are stressed [24], [25]. When placed from these environments into a selective enrichment culture their growth can be inhibited due to antibiotics or other selective compounds, so non-selective primary enrichment broths are used commonly [2], [13], [26]. Several studies have reported that using more than one type of enrichment method increases the likelihood of finding the target organism and/or revealing greater diversity [2], [27]–[29]. We used two culture methods, both based on IMS, which facilitated including a non-selective primary enrichment broth. It is likely that no culturing method is completely free of bias, and any study of this sort is limited by the media chosen for culturing. We selected three colonies for analysis from each Brilliance plate, but this provides only a small snapshot of the potential diversity present on a plate. It is possible that different and/or additional serotypes and MLVA types would have been detected if twice as many or more colonies would have been selected. The number of colonies selected is a statistical sampling and the more selected, the better the analysis. Within the logistical realities of our study three colonies was able to be managed. We chose BLEB without added supplements because it is a rich, buffered medium favoring the growth of *L. monocytogenes*. In some common *L. monocytogenes* enrichment protocols an initial 4 hour incubation in BLEB without antimicrobial supplements is used to aid in the recovery of stressed cells before the addition of antibiotics and dyes [13]. The primary enrichment temperature of 30°C is also conducive to *L. monocytogenes* and hinders some competing enterics, and some soil and water bacteria.

The strategy takes advantage of IMS beads to concentrate the *L. monocytogenes* strains for easier identification by direct plating and after selective enrichment. The IMS step was essential for detection of *L. monocytogenes* via direct plating onto Brilliance plates. Instances where the BLEB enrichment culture was plated directly onto Brilliance (or other *Listeria* selective media) without the intermediate IMS step yielded no *Listeria*-like colonies (data not shown). A classic technique, which does not use IMS or a secondary enrichment, is to concentrate an aliquot of enrichment culture by centrifugation before directly plating onto selective agar. For water samples several hundred milliliters can be filtered, and the filters placed on selective agar [30]. Moore swabs were used in the current study so that large amounts of water were not collected. Brilliance *Listeria* agar was used to select for *Listeria* spp. and differentiates pathogenic from non-pathogenic *Listeria* spp. via the clearing of lecithin due to the action phosphotidylcholine phospholipase C activity encoded by the virulence gene *plcB* [31]. We experimented with other non-colorimetric agars such as Oxford Agar, Modified Oxford Agar, and PALCAM medium, but this resulted in many false positives (data not shown). Studies have reported that while IMS increases the chances for *Listeria* detection, it does not necessarily increase the ratio of recovery for *L. monocytogenes* over other *Listeria* spp [32]. Therefore, using a colorimetric agar decreases the workload by allowing the focus to be placed on colonies that display qualities unique to virulent *Listeria* spp. (e.g., *L. monocytogenes* and *L. ivanovii*), rather than a non-colorimetric agar where colonies from all *Listeria* spp. look similar [33], [34]. The data revealed that using both direct plating of IMS beads and plating following a Fraser Broth subculture of the IMS beads revealed greater diversity among the isolates than would have resulted with one method alone. Loncarevic *et al* [35] reported a greater variety of strains of *L. monocytogenes* after direct plating of food samples compared to plating samples from selective enrichment of those foods, suggesting variable fitness of strains in selective culture.

To assess the ecology of *L. monocytogenes* it is important to determine if the two methods were enriching the same subtypes. Since serotype is not discriminating enough to measure this, MLVA was used for molecular subtyping of isolates. The MLVA protocol measured tandem repeats in 8 different loci. We altered the parameters for the Lm-32 locus to have a minimum number of 10 tandem repeats because we detected a population of isolates with strong peaks consistent with a minimum less than the previously reported 13 [15]. For the 304 isolates in this study we detected 3 different serotypes and 62 different MLVA types. A previous study of urban and natural environments in New York reported a large diversity of *Listeria* subtypes as measured by *sigB* (Sigma B) allelic types among 80 isolates of *L. monocytogenes* [5]. The more common MLVA types in the present study were detected well from both enrichment types (Figure 2). This information is critical for determining if the methods are measuring accurately the subtype diversity in the environment. Regardless, the use of two methods will reveal a greater diversity than either one alone. An example of the value of the direct plating strategy is illustrated in Figure 2 showing that strains of MLVA types 30–33, 39, and 61, which represent 10 locus differences between MLVA types 36 and 59 strains, were isolated only by direct plating. Several isolates in the present study were missing one or more of the alleles, and this might reflect absence of the allele or SNP differences causing poor primer binding. Different MVLA protocols have been described recently that may provide better discrimination in future studies [17].

Three serotypes were detected by the two methods, but the majority of the isolates were serotype 4b. While this serotype is more often implicated in human illness, surveys of agricultural environments and food processing facilities for *L. monocytogenes* result in serotype 1/2a strains isolated much more frequently than serotype 4b [36], [37]. Our results suggested that culturing with Fraser broth allowed for detection of more serotype 1/2a strains than Direct plating of the IMS beads. Previous studies indicate that serotype 1/2a strains might outcompete serotype 4b strains in some types of selective enrichment culture [8], but another study using BLEB with some of the supplements present in Fraser Broth reported no bias based on the serotype difference [11]. In a study of Canadian surface waters where direct-plating of water onto selective medium and enrichment methods were compared different serotype groupings were isolated based on the culture method used [30]. The results of an earlier Canadian watershed study reported that >50% of the isolates from 314 water samples over 5 months were of serotype 1/2a and 3a, and 32% of the isolates were serotype 4 strains [1]. This study utilized selective primary and secondary enrichments before plating on a selective and differential medium. The number of serotype 4b isolates observed in the present study is inconsistent with these results, and suggest that serotype 4b was the predominant serotype in these watershed samples and/or that serotype 4b strains were less likely to be isolated than 1/2a or 3a strains by the selective methods used in the Canadian study.

While the majority of isolates were of serotype 4b, they belonged to many MLVA types, indicating a wide diversity of genetic subtypes within serotypes and in the watershed. This could indicate microevolution of *L. monocytogenes* occurring in the watershed environment or multiple incidents of contamination from other point sources (e.g. livestock or wildlife) representing diverse genetic subtypes. Other studies have isolated a more diverse set of *L. monocytogenes* subtypes from direct plating as compared to standard, selective enrichment culture [35].

There are few studies on the ecology of *L. monocytogenes* in watersheds near agricultural environments. We determined the types of isolates obtained through two types of enrichment in a preliminary study of *L. monocytogenes* in an agricultural watershed. There is a concern that wildlife may carry pathogens from contaminated watersheds to fields, and/or sections of agricultural land flooded after heavy rains may be contaminated. The present study indicates that there is much more information to be obtained about the ecology of *L. monocytogenes* in this leafy greens production watershed environment. The enrichment methods tested in this study can provide in subsequent studies a diverse collection of isolates to aid in determining the incidence and ecology of the pathogen outside of the host. Surveys of similar environments in other regions could assist in determining the predominant subtypes and whether subtypes differ by location.

## Supporting Information

Table S1 **Characteristics of isolates from Watershed samples.**
(XLSX)
